# Gut microbiota therapy for chronic kidney disease

**DOI:** 10.3389/fimmu.2025.1660226

**Published:** 2025-09-10

**Authors:** Chunguang Liu, Junhong Wang, Lei Lei, Liping Li, Xingxing Yuan

**Affiliations:** ^1^ Department of Nephrology, Heilongjiang Academy of Traditional Chinese Medicine, Harbin, China; ^2^ Department of Internal Medicine, Harbin Hospital of Traditional Chinese Medicine, Harbin, China; ^3^ Department of Gastroenterology, Heilongjiang Academy of traditional Chinese medicine, Harbin, China

**Keywords:** gut microbiota, chronic kidney disease, therapeutic interventions, fecal microbiota transplantation, precision-based prevention

## Abstract

Chronic kidney disease (CKD), affecting 13% of the global population, is increasingly linked to gut microbiota dysbiosis, a condition driven by uremic toxins accumulation, metabolic alterations, and dietary factors. This mini review explores gut microbiota modulation as a therapeutic strategy to alleviate CKD symptoms, focusing on interventions that target gut microbiota composition and function. Prebiotics, such as resistant starch, have been shown to lower uremic toxins and reduce inflammation, while dietary adjustments, including low-protein and gluten-free diets, modulate microbial diversity and improve renal biomarkers. Fecal microbiota transplantation (FMT), which stabilizes creatinine levels and shifts gut microbiota toward beneficial taxa, represents another promising approach. However, limitations persist: synbiotics, which often induce gut microbiota shifts, frequently lack clinical impact; probiotics, which enhance glucose control and oxidative stress mitigation, exhibit variable efficacy; and interventions such as propolis or cranberry extract, which have been tested, prove ineffective. The causal relationship between gut microbiota dysbiosis and CKD progression, which remains unclear, is further complicated by methodological heterogeneity across studies. Emerging strategies, including phage therapy and artificial intelligence-driven multi-omics integration, which hold significant promise, require further validation. Future research must prioritize longitudinal studies, maternal gut microbiota optimization, and personalized approaches, which are essential for advancing CKD management. While gut microbiota modulations hold therapeutic potential, translating these findings into clinical practice demands rigorous trials to address inconsistencies and establish mechanistic links, ultimately shifting CKD management from reactive treatment to precision-based prevention.

## Introduction

Chronic kidney disease (CKD), affecting approximately 13% of the global population, represents a significant public health burden characterized by progressive loss of renal function (1). A hallmark feature of CKD is profound gut microbiota dysbiosis, characterized by shifts such as increased *Enterobacteriaceae* and *Streptococcus*, and decreased beneficial taxa like *Prevotella* and *Roseburia* (2). This dysbiosis is driven by uremic toxin accumulation, metabolic acidosis, dietary restrictions, and frequent antibiotic use, disrupting intestinal barrier integrity and promoting inflammation (3, 4). Critically, this altered microbial ecology generates pathogenic metabolites, including gut-derived uremic toxins like indoxyl sulfate, p-cresyl sulfate, and trimethylamine-N-oxide (TMAO) (4, 5). Elevated TMAO levels correlate with inflammation, reduced glomerular filtration rate (GFR), and increased mortality in CKD patients (6, 7), while reduced short-chain fatty acid (SCFA) production by diminished commensal bacteria further exacerbates renal injury and systemic inflammation (8, 9). These microbial metabolites directly contribute to CKD progression and associated complications (10). Targeting this dysbiotic gut environment offers a promising therapeutic avenue. Emerging evidence highlights Traditional Chinese Medicine (TCM) as a potent modulator of the gut-kidney axis. TCM formulations like Yi-Shen-Hua-Shi granules and Zicuiyin decoction mitigate proteinuria, preserve renal function (eGFR), and ameliorate CKD progression by specifically reversing gut dysbiosis, enriching beneficial genera (*Faecalibacterium*, *Lachnoclostridium*, *Lactobacillaceae*) and suppressing pathogenic bacteria such as *Clostridium innocuum*, *Enterobacteriales* (11, 12).

CKD is globally prevalent, with gut microbiota dysbiosis increasingly implicated in its pathogenesis. Bibliometric analysis confirms intense research focus on microbiota-CKD interactions, particularly regarding disease mechanisms, probiotic therapies, and microbial metabolites (13). Specific microbial alterations, such as depletion of *Lactobacillus johnsonii*, correlate strongly with CKD progression and uremic toxin accumulation. Restoring this bacterium ameliorates renal injury via indole-3-aldehyde-mediated aryl hydrocarbon receptor signaling (14). In diabetic kidney disease (DKD), gut-derived metabolites critically influence pathophysiology through molecular pathways affecting inflammation, fibrosis, and metabolic homeostasis (15). These findings highlight microbiota modulation, via probiotics, metabolites, or dietary interventions as a promising therapeutic strategy for CKD management.

This modulation reduces uremic toxin burden, strengthens intestinal barrier function, and dampens inflammation, positioning TCM as a key strategy for microbiota-targeted CKD management. The primary aim of this mini-review is to evaluate progress in gut microbiota modulation for improving CKD outcomes. While existing systematic reviews and meta-analyses are limited by narrow sampling frames focused on contemporary trials, this review adopts a distinct approach by exclusively for clinical trials and randomized controlled trials to strengthen the evidence base. By synthesizing current findings, this review provides a comprehensive perspective on the role of gut microbiota in improving CKD management and patient longevity.

## Gut microbiota alterations in CKD

CKD is characterized by significant alterations in gut microbiota composition, including an increased abundance of *Streptococcaceae*, *Enterobacteriaceae*, and *Streptococcus*, alongside reduced levels of *Prevotellaceae*, *Prevotella 9*, *Prevotella*, and *Roseburia* (2). Similarly, patients with kidney stones also exhibit distinct microbial variations, such as shifts in the *Lachnospiraceae NK4A136* group, *Bacteroides*, *Ruminiclostridium 5* group, *Enterobacter*, *Dorea*, and *Christensenellaceae* (16). In DKD, the gut microbiota profile is marked by enriched *Escherichia* and *Hungatella genera* and reduced butyrate-producing bacteria (8), as well as increased *Citrobacter* and *Klebsiella genera* with decreased *Roseburia*, highlighting potential targets for therapeutic intervention (17). Notably, these diabetic microvascular complications are marked by reduced SCFA-producing bacteria and diminished alpha diversity, reinforcing the therapeutic potential of gut microbiota modulation across kidney diseases (9).

Patients with idiopathic membranous nephropathy exhibit elevated *Proteobacteria* and reduced *Lachnospira*, highlighting key gut microbiota alterations (18). In lupus nephritis, decreased inflammatory indicators and *Firmicutes*/*Bacteroidetes* ratios, coupled with intestinal barrier dysfunction, serve as pathogenic markers (19). Metabolically, reduced saccharolytic bacteria and increased nitrogen-compound fermenters are linked to circulating uremic toxins in CKD (10). IgA nephropathy is associated with *Escherichia-Shigella* expansion, suggesting novel diagnostic and therapeutic targets (20). By enhancing intestinal barrier function to prevent hepatotoxic metabolite formation and modulating immune responses, microbiota-targeted therapies may improve non-alcoholic fatty liver disease (NAFLD) (21). Dietary modifications, alongside lifestyle changes, represent preventive strategies for NAFLD, thereby mitigating CKD risk factors (22).

Efforts to elucidate the causal and correlative effects of gut microbiota in CKD have identified distinct microbial species and families rather than overall diversity during low-protein diet interventions (23). Dietary fiber supplementation reduces creatinine and serum urea levels, underscoring the role of uremic toxins in CKD progression (24). Resistant starch, particularly type 2 resistant starch, lowers uremic toxins and inflammation, improving renal function in patients with CKD and enhancing residual renal function in maintenance hemodialysis patients (25). Anthocyanin degradation into phenolic acids and colonic metabolites regulates biological activities, including CKD amelioration, when systemically accumulated (26). Synbiotic interventions reduce oxidative stress, inflammation, and uremic toxins in hemodialysis patients, though their efficacy in CKD management remains insufficient (27).

**Figure 1:**
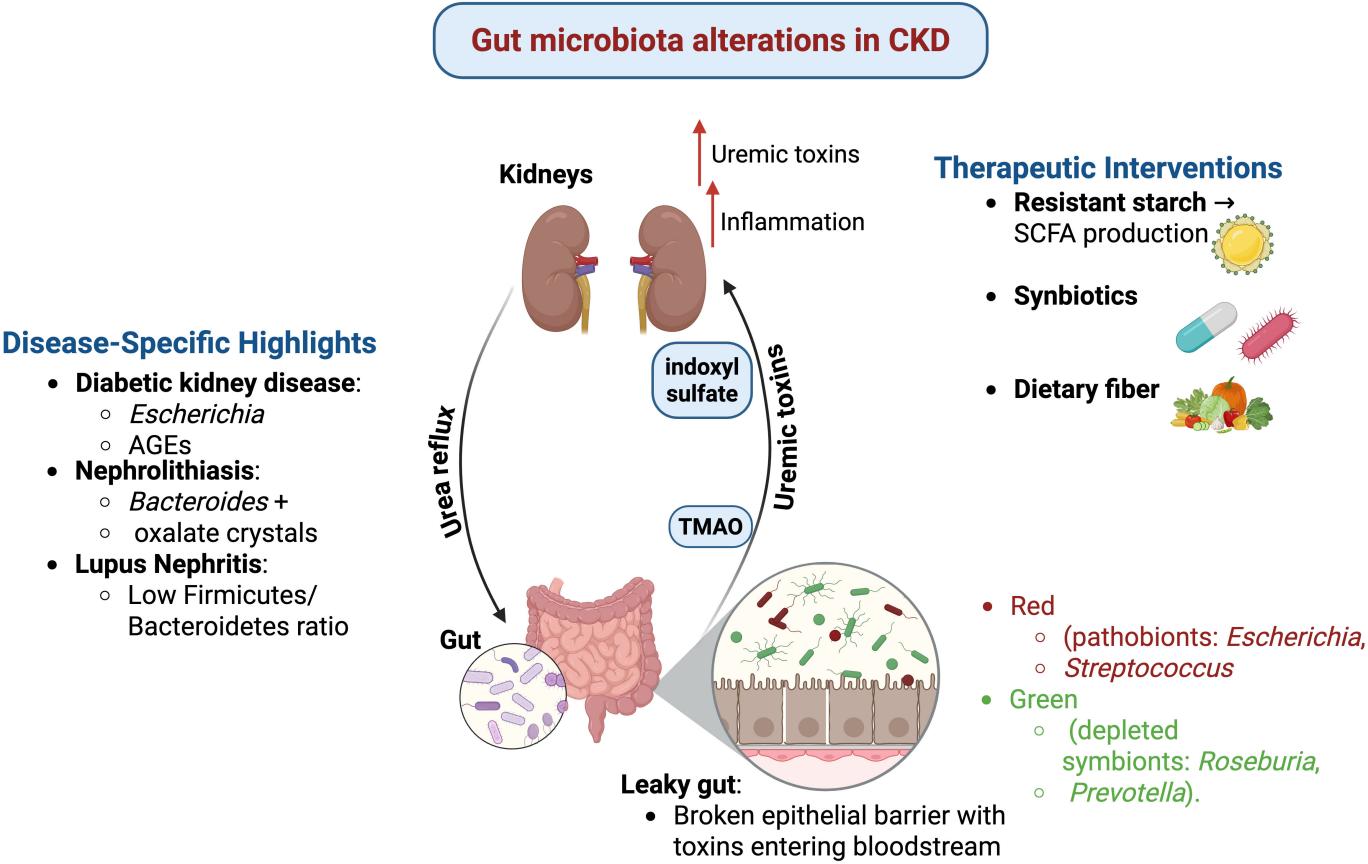
Gut microbiota alterations in CKD. CKD is associated with gut dysbiosis, leading to increased production of uremic toxins (e.g., indoxyl sulfate, TMAO) that exacerbate kidney inflammation and dysfunction. Urea reflux from kidneys to the gut further disrupts microbial balance, resulting in a “leaky gut,” where toxins cross the impaired epithelial barrier into circulation. Disease-specific microbial signatures include Escherichia and advanced glycation end products (AGEs) in diabetic kidney disease, Bacteroides and oxalate crystals in nephrolithiasis, and a low Firmicutes/Bacteroidetes ratio in lupus nephritis. Pathobionts (e.g., Escherichia, Streptococcus) increase, while beneficial symbionts (e.g., Roseburia, Prevotella) are depleted. Therapeutic interventions such as resistant starch (enhancing SCFA production), synbiotics, and dietary fiber aim to restore microbial balance and reduce uremic toxin burden.

The unique gut microbiota profile in kidney stone patients suggests that dietary adjustments and personalized therapies, such as synbiotics, may restore eubiosis and prevent stone formation/recurrence (28). Synbiotics also mitigate uremic solute production, oxidative stress, and systemic inflammation (29). However, while synbiotics increase Bifidobacterium abundance, their clinical efficacy in CKD management remains limited (30). Longer-term supplementation may improve inflammatory and renal indices in CKD, though large-scale trials are needed to validate these findings (31). TCM interventions show potential for CKD improvement, but efficacy validation, safety concerns, and barriers to international collaboration hinder progress (32, 33). Disorders of gut-derived metabolites, including p-cresyl sulfate, indoxyl sulfate, indole-3-acetic acid (IAA), and indole-3-aldehyde (IAld), drive kidney injury in AKI and CKD by activating aryl hydrocarbon receptor (AhR) pathways and promoting inflammation/fibrosis (34). Depleted *Lactobacillus* species (*L. johnsonii*) reduce protective IAld, elevating toxic IAA and indoxyl sulfate, which accelerate renal damage (35). Mendelian randomization confirms causal links: specific microbiota (*Bacteroides*) perturb metabolites like glycocholenate sulfate and α-ketoglutarate, directly influencing diabetic nephropathy progression (36, 37). Restoring probiotic balance (*Lactobacillus*) normalizes tryptophan-derived metabolites, inhibiting AhR and offering therapeutic strategies for kidney diseases (34, 35) (**Figure 1**).

## Gut microbiota modulation for improved kidney functions

### Synbiotics, probiotics, and prebiotics supplementation

Interventional studies have demonstrated the potential of gut microbiota modulation in improving kidney function through synbiotics, probiotics, and prebiotics supplementation. Synbiotic formulations containing *Bifidobacterium lactis*, *Lactobacillus casei*, and *Lactobacillus acidophilus* have been shown to reduce uremic toxins, lower indoxyl sulfate serum levels, and mitigate microinflammation in patients with CKD. These interventions modulate gut microbiota composition toward beneficial genera such as *Subdoligranulum*, *Bifidobacteria*, and *Lactobacillus*, thereby enhancing estimated glomerular filtration rate (eGFR) and reducing high-sensitivity C-reactive protein levels (38). Synbiotic meals have also been effective in lowering uremic toxins in hemodialysis patients (39) and reducing plasma p-cresol levels in kidney transplant recipients, highlighting their therapeutic relevance (40). Additionally, synbiotics improve serum brain-derived neurotrophic factor levels and alleviate depression symptoms in hemodialysis patients (41). Combined treatment with synbiotics and divinylbenzene-polyvinyl pyrrolidone hemodialysis has been shown to reduce indoxyl sulfate and p-cresyl sulfate across dialysis modalities, validating multi-interventional strategies (42).

Probiotic supplementation has been associated with improved glucose homeostasis, reduced oxidative stress, and decreased inflammation in patients with diabetic hemodialysis (43). Prebiotics and probiotics have been shown to increase T-reg cells (CD4+/CD25+/FOXp3+) and *Lactobacillus* abundance while reducing relapse rates in idiopathic nephrotic syndrome (44). Inulin-type fructans enhance gut microbiota-generated indole production in peritoneal dialysis patients (45), although synbiotics have been reported to elevate both parathyroid hormone and indoxyl sulfate levels (46). Probiotics also reduce uremic solutes such as 1-methylinosine, 3-guanidinopropionic acid, indole-3-acetic acid-O-glucuronide, while shifting gut microbiota composition and diversity (47).

The prebiotic β-glucan has been shown to lower gut microbiota-induced uremic toxins, irrespective of BMI, triglyceride levels, or HDL status, marked by increased *Bacteroides* and *Prevotella* (48). Prebiotic fructooligosaccharide (FOS) regulates IL-6 and preserves endothelial function in CKD patients with endothelial damage (49). Supplementation with *Bifidobacterium longum* and sorghum flakes reduces BMI, improves gastrointestinal symptoms, enhances SCFA synthesis, boosts Chao1 diversity, and lowers uremic toxins in CKD (50). Probiotic cocktails containing *Lactobacillus reuteri* and *Bifidobacterium longum* reduce microbial toxins, complementing diuretic and antihypertensive therapies. Low-protein diet further modulates proatherogenic toxins and microbiota in CKD (51). Probiotics also elevate *Bifidobacterium* spp., *Akkermansia muciniphila*, and *Barnesiella intestinihominis*, offering clinical benefits in metastatic renal cell carcinoma (52).

### Dietary supplementation

Dietary supplementation plays a critical role in gut microbiota modulation and kidney disease management. Diet quality influences uremic toxin levels, gut microbiota composition, diversity, and functionality in adult CKD patients. Optimizing the protein-to-fiber ratio to favor *Oscillospirales* may benefit CKD patients, while avoiding discretionary foods, artificial sweeteners, sweet desserts, and potatoes supports *Prevotella* species (53). CKD patients on low-protein diet exhibit enriched ketone bodies, glutathione metabolism, and D-alanine as bacterial gene markers. CKD-low-protein diet also increases glyco λ-muricholic acid, secondary bile acids, and butanoate metabolism, alongside reduced SCFA serum levels and butyrate-producing bacteria, revealing gut microbiota adaptations to dietary protein (54). Gluten-free/dairy-free diets elevate T regulatory/T helper 17 cell ratios and shift gut microbiota favorably in children with steroid-resistant nephrotic syndrome (SRNS) (55).

RS supplementation reduces platelet-derived growth factor (PDGF), regulated upon activation, normal T cell expressed and secreted (RANTES) [also known as CCL5], and interferon-inducible protein 10 (IP-10) [also known as CXCL10] in CKD (56). Resistant starch modulates plasma indole-3-acetic acid and aryl hydrocarbon receptor mRNA expression in hemodialysis patients (57). Prebiotic-resistant starch increases fiber intake while lowering IL-6, thiobarbituric acid reactive substances, and indoxyl sulfate (41). High-amylose maize resistant starch elevates *Faecalibacterium* in ESRD patients (58), and type 2 resistant starch promotes SCFA-producing bacteria, positioning it as a key gut microbiota modulation strategy for CKD (59).

Dietary fiber enhances renal anemia in ESRD by increasing serum butyric acid, hemoglobin, ferritin, Fe^2+^, *Lactobacillus*, *Bifidobacterium adolescentis*, and *Lactobacillaceae* (38). Curcumin also reduces pro-inflammatory mediators (IFN-γ, CCL-2, IL-4) and lipid peroxidation while expanding *Lachnoclostridium* and *Lactobacillaceae* over *Escherichia-Shigella* in CKD patients (60). Curcumin also lowers p-cresyl sulfate plasma levels in hemodialysis via gut microbiota modulation (61). Fecal microbiota transplantation (FMT) stabilizes urea nitrogen and serum creatinine, slows disease progression, and shifts gut microbiota toward *Roseburia* spp., *Proteobacteria*, and *Bacteroidetes* with reduced *Actinobacteria* and *Firmicutes* (62, 63). ACEI/ARB therapy combined with FMT reduces urinary protein in IgA nephropathy patients, correlating with *Phocaeicola_dorei*, *Prevotella_copri*, *Bacteroides_uniformis*, and altered metabolites including serotonin, phosphatidylcholine, fumagillin (64).

### Traditional Chinese medicine

TCM has also shown promise in gut microbiota modulation and kidney disease treatment. Yi-Shen-Hua-Shi granules mitigate proteinuria and reverse gut microbiota dysbiosis in CKD by increasing *Faecalibacterium*, *Lachnoclostridium*, *Sutterella*, and *Lachnospiraceae* while reducing *Clostridium innocuum* and *Eggerthella* (12). Zicuiyin decoction preserves kidney function in patients with gut microbiota dysbiosis and declining eGFR, promoting *Lactobacillaceae* and *Prevotellaceae* whilesuppressing *Clostridiaceae*, *Enterobacteriales*, and *Micrococcaceae* (11). Qushi Huayu formula alleviates NAFLD by lowering liver enzymes, fat content, and phenylalanine/tyrosine, while increasing p-hydroxyphenylacetic acid (65). The spleen-strengthening and liver-draining formula improves glucolipid metabolism and liver function in NAFLD via *Ruminococcus*, *Coprococcus*, and *Lachnospiraceae_NK4A136* (66). Fushen granule enriches gut microbiota with *Megamonas*, *Rothia*, and *Bacteroides*, improving quality of life and nutritional status in peritoneal dialysis-related peritonitis (67).

**Figure 2:**
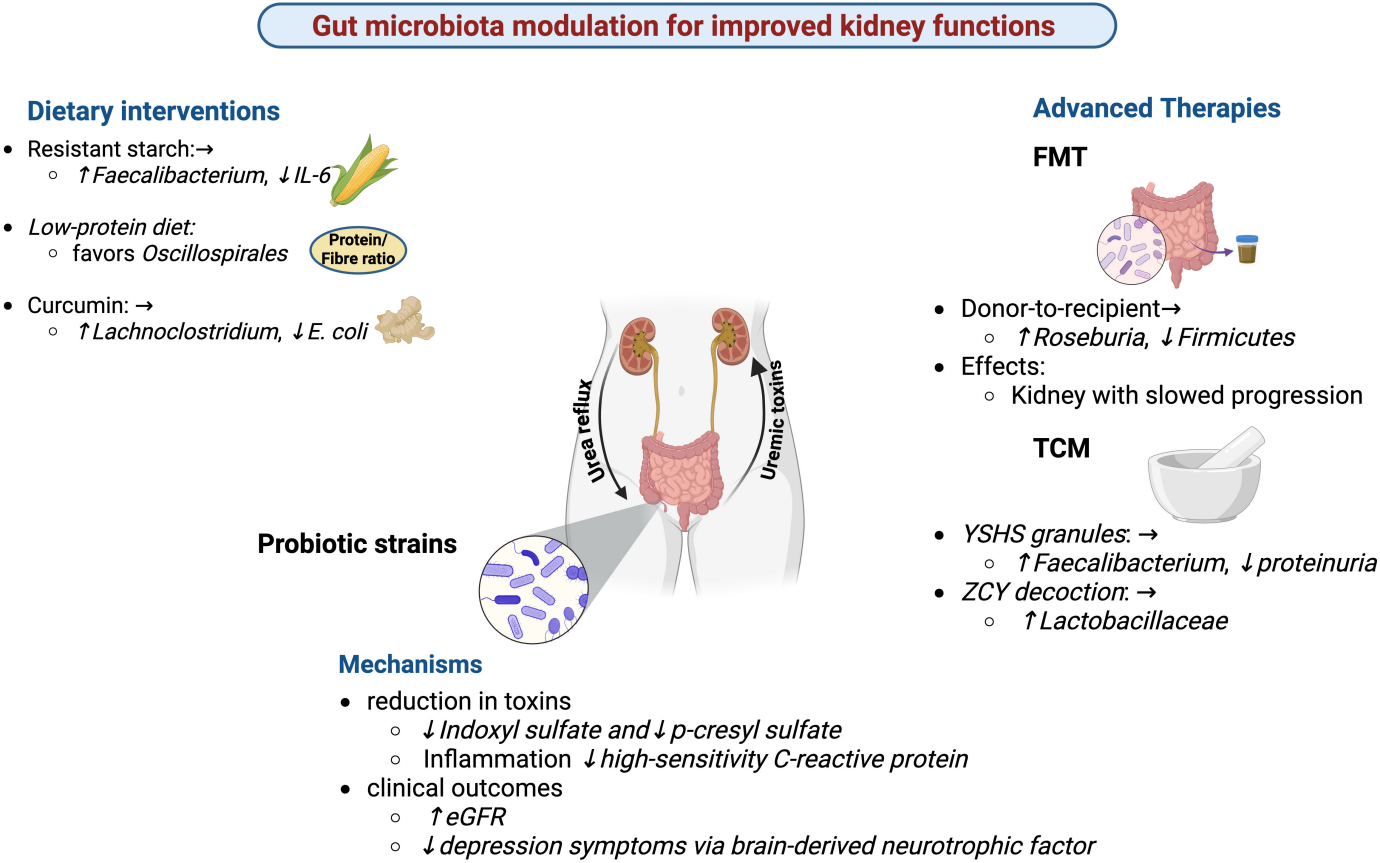
Gut microbiota modulation for improved kidney functions. Dietary interventions (resistant starch, low-protein diet, and curcumin), probiotics, and advanced therapies (fecal microbiota transplantation and traditional Chinese medicine) modulate gut microbial composition in chronic kidney disease (CKD). These approaches enhance beneficial taxa (e.g., Faecalibacterium, Roseburia, Lactobacillaceae), suppress harmful bacteria (e.g., E. coli), reduce uremic toxins (indoxyl sulfate, p-cresyl sulfate), and lower inflammation. Improved microbial balance is associated with better clinical outcomes, including increased eGFR, reduced proteinuria, and alleviation of depression symptoms.

TCM alleviates AKI and CKD by modulating gut microbiota and metabolites. TCM formulations enrich beneficial taxa (*Lactobacillaceae*, *Prevotellaceae*) while suppressing pathogens (*Enterobacteriaceae*), enhancing SCFA production and reducing uremic toxins like indoxyl sulfate and indole-3-acetic acid (68–70). This rebalancing strengthens intestinal barrier integrity, inhibits oxidative stress, and downregulates aryl hydrocarbon receptor (AhR)-mediated inflammation and renal fibrosis (70, 71). For instance, mild-natured sweet-flavored TCMs elevate SCFA-producing *Ruminococcus* and *Bacteroides*, lowering p-cresyl sulfate and lipopolysaccharides (68, 69). Clinical studies confirm TCM’s efficacy in reducing proteinuria and slowing CKD progression via microbiota-metabolite crosstalk (71, 72) (**Figure 2**).

## Limitations and future prospects

Despite its microbial richness, propolis intervention did not alter plasma levels of uremic toxins such as indole-3 acetic acid, p-cresyl sulfate, and indoxyl sulfate, nor did it significantly change gut microbiota composition (73). Similarly, curcuminoid supplementation attenuated lipid peroxidation and reduced plasma p-cresyl sulfate and malondialdehyde in CKD patients undergoing peritoneal dialysis, but it showed no significant effects on plasma cytokines, Nrf2 mRNA expression, protein thiols, HO-1, or NF-κB (74). Synbiotics, while favorably modifying gut microbiota and reducing serum p-cresyl sulfate, failed to significantly lower serum indoxyl sulfate, suggesting that gut microbiota shifts alone may lack clinical relevance (75). Furthermore, 12-week synbiotic supplementation demonstrated no effect on nephropathy, and although synbiotics altered gut microbiota (*Bifidobacterium* and *Blautia* spp.) and reduced eGFR, further studies are needed to clarify their impact on kidney function (76, 77).

Probiotics also showed limited efficacy. They did not alter plasma TMAO levels in hemodialysis patients or SCFA levels in peritoneal dialysis patients, despite gut microbiota changes (78, 79). Trans-resveratrol supplementation did not reduce uremic toxins, despite a negative correlation with GFR (80). Similarly, *CBM588* bifidogenic bacteria improved clinical activity but exerted no protective effects in metastatic renal cell carcinoma patients receiving nivolumab and cabozantinib (81). Unripe banana flour intervention did not improve serum biomarkers of kidney function, and cranberry dry extract failed to reduce uremic toxins or plasma lipopolysaccharides in non-dialysis CKD patients (82, 83). Inulin-type fructans as prebiotics did not alter major components in ESRD, despite favorable arsenic levels (84). A probiotic cocktail containing *Streptococcus thermophilus*, *Lactobacillus acidophilus*, and *Bifidobacterium longum* showed no benefit in hemodialysis patients (85), and physical exercise did not modulate gut microbiota-derived uremic toxins in hemodialysis (86). Short-term rifaximin treatment failed to reduce gut-derived cardiovascular toxins or inflammatory cytokines in CKD (87).

The causal relationship between gut microbiota dysbiosis and CKD remains unclear, though renal disease and its treatments likely influence microbiota (88). Colonic dialysis mitigated gut microbiota dysbiosis and protected renal function in pre-dialysis CKD (89). However, sucroferric oxyhydroxide and calcium acetate supplementation did not modify gut microbiota in CKD patients (90). Time-restricted feeding improved renal function by favorably shifting gut microbiota and regulating body weight, fat-free mass, body fat mass, and body water (91). Dietary restriction altered gut microbiota in peritoneal dialysis patients via advanced glycation end products, and oral vancomycin combined with underfeeding may offer therapeutic potential by modulating gut microbiota and nutrient absorption in CKD (92, 93).

Adults with idiopathic nephrotic syndrome exhibit gut microbiota alterations correlated with clinical parameters, informing novel therapeutic and diagnostic strategies (94). High-quality probiotics should be studied alongside gut microbiota dysbiosis, iron status, inflammatory indices, and serum iPTH stabilization in CKD patients (95). Gut microbiota-dependent TMAO correlates with long-term all-cause mortality in CKD (96). Short-term metformin therapy with prebiotic fiber showed tolerable clinical benefits in youth with type 2 diabetes via microbial shifts (97). FOS may reduce free p-cresyl sulfate and total serum levels in nondiabetic CKD, though secondary outcomes were unchanged, warranting further studies (98).

## Conclusion

Interventional studies show that gut microbiota modulation via synbiotics, probiotics, and prebiotics reduces uremic toxins, inflammation, and oxidative stress in CKD, improving renal function and glycemic control. Dietary strategies like resistant starch and curcumin enhance microbial diversity, increase SCFA production, and strengthen intestinal barrier integrity. TCM reverse gut microbiota dysbiosis and alleviate proteinuria. However, limitations exist. Synbiotics often fail to lower indoxyl sulfate, probiotics show inconsistent affect TMAO and SCFA levels, and interventions like propolis or cranberry extract lack efficacy. FMT and dietary adjustments stabilize renal biomarkers and modulate microbial ecology, yet causal links between gut microbiota and CKD remain unclear. Emerging therapies, including phage therapy and artificial intelligence-driven multi-omics integration, hold promise but require validation. Future research must prioritize longitudinal studies, maternal gut microbiota optimization, and personalized approaches to translate gut microbiota modulation into clinically meaningful renal health outcomes.
